# Effects of feeding lubabegron on gas emissions, growth performance, and carcass characteristics of beef cattle housed in small-pen environmentally monitored enclosures during the last 3 mo of the finishing period

**DOI:** 10.1093/jas/skab338

**Published:** 2021-11-27

**Authors:** J Scott Teeter, Samantha J Werth, Sandra L Gruber, John C Kube, Jacob A Hagenmaier, Janet B Allen, Cory T Herr, Michael S Brown, Dustin Boler, Anna C Dilger, Yongjing Zhao, Yuee Pan, Frank M Mitloehner

**Affiliations:** 1 Elanco, Greenfield, IN 46140, USA; 2 Department of Animal Science, University of California, Davis, CA 95616, USA; 3 Department of Animal Sciences, University of Illinois, Urbana-Champaign, Urbana, IL 61801, USA

**Keywords:** ammonia gas emissions, carcass characteristics, environment, feedlot cattle, lubabegron, tenderness

## Abstract

The development of technologies that promote environmental stewardship while maintaining or improving the efficiency of food animal production is essential to the sustainability of producing a food supply to meet the demands of a growing population. As such, Elanco (Greenfield, IN) pursued an environmental indication for a selective β-modulator (lubabegron; **LUB**). LUB was recently approved by the United States Food and Drug Administration (**FDA**) to be fed to feedlot cattle during the last 14 to 91 d of the feeding period for reductions in gas emissions/kg of unshrunk final BW and HCW. A 4 × 2 factorial arrangement of treatments was used with the factors of dose (0.0, 1.38, 5.5, or 22.0 mg·kg^−1^ DM basis) and sex (steers or heifers). Three 91-d cycles were conducted (112 cattle/cycle) with each dose × sex combination being represented by a single cattle pen enclosure (**CPE**; 14 cattle/CPE) resulting in a total of 168 steers and 168 heifers (*n* = 6 replicates/dose). There were no interactions observed between dose and sex for any variable measured in the study (*P* ≥ 0.063). Five gases were evaluated for all pens based on CPE concentrations relative to ambient air: NH_3_, CH_4_, N_2_O, H_2_S, and CO_2_. Cumulative NH_3_ gas emissions were reduced by feeding cattle 5.5 and 22.0 mg·kg^−1^ LUB (*P* ≤ 0.023) and tended (*P* = 0.076) to be lower for the cattle fed 1.38 mg·kg^−1^ LUB compared with the negative controls (**CON**). The cumulative NH_3_ gas emission reductions of 960 to 1032 g, coupled with HCW increases (*P* ≤ 0.019) of 15 to 16 kg for all LUB doses vs. CON, led to reductions in NH_3_ gas emissions/kg HCW for all three LUB treatments (*P* ≤ 0.004). Similar to HCW, reductions in NH_3_ gas emissions/kg of unshrunk final BW were observed for all LUB doses (*P* ≤ 0.009) and were attributable to both decreases in NH_3_ gas emissions and numerical increases in BW. Dose had no effect on cumulative emissions or emissions standardized by BW or HCW for the other four gases (*P* ≥ 0.268). LUB is a novel tool to reduce emissions of NH_3_ gas per kilogram of unshrunk live BW and hot carcass weight.

## Introduction

Traditionally, the impact of livestock production management practices on the environment has been evaluated using primarily life cycle assessments (Clark and Tilman, 2017; Rotz et al., 2019). Less work has been conducted measuring direct changes to the environment when tested in controlled experimental settings. Furthermore, no clinical registration programs for products approved by the United States Food and Drug Administration (**FDA**) have targeted reductions in specific gas analytes of environmental concern. Consumer attention to emissions from modern food production systems has intensified, and compelled Elanco Animal Health (Greenfield, IN) to pursue a label indication for the reduction of emissions for a new feed additive containing the active pharmaceutical ingredient lubabegron (**LUB**; experior).

LUB is a selective β-modulator (**SβM**) which selectively binds to the β-adrenergic receptor and has agonistic properties at the β _3_-receptor subtype and antagonistic properties at β _1_- and β _2_-receptor subtypes in cattle (Dilger et al., 2021), and as such, is classified by the Center of Veterinary Medicine (**CVM**) as a ‘beta-adrenergic agonist/antagonist’ (FDA, 2018). This pharmacodynamic profile differentiates LUB from the β-ligands historically used in livestock species, as their apparent mode of action is predominately through agonistic behavior at either the β _1_- or the β _2_-receptor subtype. LUB’s affinity for the β _3_-receptor and the ability to antagonistically bind at the β _1_- and β _2_-receptors distinguish it as a novel technology and warranted evaluation for reducing the environmental impact of beef production. Additionally, LUB selectively binds to β-adrenergic receptors (binding affinity observed at ≤0.5 nM) and has low binding affinity for non-β-adrenergic receptors (i.e., no affinity observed at >300 nM for muscarinic, 5-HT_2_, dopamine D_1_ and D_2_, α _1_- and α _2_-adrenergic, benzodiazepine, histamine H_1_, or GABA_A_ receptors; FDA, 2018). Because of its selectivity and modulating characteristics, LUB can simply and accurately be described as a selective β-modulator, SβM (Dilger et al., 2021).

The current study represents a portion of the data submitted to the FDA for LUB approval. Two studies were conducted to provide evidence of clinical effectiveness. One study was 91-d duration and is described here and the other was a 14-d duration study (FDA, 2018). These studies were submitted to the FDA to support an indication for a reduction in ammonia gas emissions per unit of unshrunk BW and HCW when LUB is administered to beef steers and heifers during the last 14 to 91 d on feed.

A postapproval study (Kube et al., 2021) was conducted to determine if the results from the clinical effectiveness studies were applicable to a commercial feedyard setting when LUB was fed at the low, middle, and high approved inclusion rates (1.5, 3.5, and 5.5 mg·kg^−1^ DM) to steers (60 steers/pen; 12 pens/treatment) the last 56 d of the finishing period. Calculated cumulative ammonia gas emissions (Brown et al., 2019) were decreased 85 to 708 g·animal^−1^ and LUB increased ADG by 0.19 to 0.26 kg·d^−1^, HCW by 11.3 to 17.1 kg, and G:F by 0.015 to 0.020 units.

## Materials and Methods

The objective of the current study was to support substantial evidence of effectiveness for a new animal drug; as such, CVM Guidance for the Industry #215 (FDA, 2011) was followed to ensure the CVM fundamentally agreed with the design, execution, and analyses proposed in the study protocol (“protocol concurrence”). This study was conducted in accordance with the Good Clinical Practice standards of the U.S. Food and Drug Administration (FDA, 2015), and the procedures outlined were approved by the University of California-Davis Animal Care and Use Committee (Protocol #17063).

### Experimental design and treatments

A randomized complete block design was used to evaluate the effect of LUB on gas emissions over a 91-d period using 336 beef cattle (BW = 453 ± 34.5 kg) housed in cattle pen enclosures (**CPE**). Four LUB treatments were included in the study based on dose: 0.0 (**CON**), 1.38, 5.5, and 22.0 (mg·kg^−1^ of DM). Because there was a limited number of CPE (*n* = 8), three sequential cycles (blocks) were required to generate 6 replicates of each dose × sex combination. As such, 112 cattle (56 steers and 56 heifers) were housed concurrently within each cycle across the eight CPE (14 cattle/CPE), with each dose × sex combination being represented by a single CPE/cycle. To assure different frame sizes of cattle were represented, cattle in cycles one and three were large-frame Continental crossbreds, whereas cattle in cycle 2 were medium-frame British crossbreds. Based on details provided in the National Research Council report on air emissions (NRC, 2003), four gases of greatest importance to animal feeding operations were selected to be measured, including ammonia (**NH**_**3**_), hydrogen sulfide (**H**_**2**_**S**), as well as the greenhouse gases nitrous oxide (**N**_**2**_**O**) and methane (**CH**_**4**_). In addition to these four, carbon dioxide (**CO**_**2**_) was also measured because of its importance as a greenhouse gas. Response variables of primary interest were the ratios of cumulative emissions to unshrunk final BW and HCW (g/kg BW and g/kg HCW, respectively) for each of these five gases.

### Study timeline and treatment allocation

Treatment administration for the three cycles occurred from April through July, August through November, and December through March of 2014 and 2015, respectively. Four weeks before beginning treatments for each cycle (day −28), up to 145 cattle were sourced from a common origin and transported to the study site at the University of California-Davis to be group-housed in single-sexed outdoor pens. The presence of growth-promoting implants was assessed, and any preexisting implants were excised before shipment to the study site to ensure they had been implant-free for a minimum of 28 d before treatments began on day 0. No additional implants were used in these cattle, and they were considered nonimplanted.

On day −8, cattle were screened for abnormal health conditions by a veterinarian and ranked by BW to identify the 56 eligible cattle within each sex that provided the narrowest weight range. The following day (day −7), the 56 cattle selected for study enrollment within each sex were grouped into sets of four consecutively weight-ranked cattle. Cattle were then randomly allocated to their respective treatment within each weight group and transferred into the CPE. The CPE were randomly assigned to sex and dose treatment before each cycle, and all study personnel were blinded to treatments throughout the duration of the study. All cattle were fed the negative control basal finishing diet (Table 1) for 1 wk (days −7 to −1) after being placed into the CPE to allow for acclimation before beginning treatments.

**Table 1.** Ingredient composition (DM basis) and analyzed nutrient content of the finishing diet fed during the 91-d treatment phase^1^

Ingredient	% of DM
Ground corn^2^	2.5
Dried distiller’s grains with solubles	10.0
Steam-flaked corn	63.5
Tallow	3.0
Cane molasses	6.0
Alfalfa	6.0
Wheat straw	6.0
Limestone	1.4
Urea	1.1
Salt	0.3
Trace minerals^3^	0.2
Total	100.0
Analyzed nutrient content, DM basis	
DM	76.5
CP, % of DM	14.2
Ca, % of DM	0.66
P, % of DM	0.31
Calculated RDP^4^, % of DM	9.34
Calculated RUP^4^, % of DM	4.86
Calculated NE_m_, Mcal·kg^−1^ DM	2.21
Calculated NE_g_, Mcal·kg^−1^ DM	1.54

^1^Water was included at 7.5% of as-fed feed to reduce likelihood of segregation of ingredients within the type C feed.

^2^Ground corn was fed without LUB in the negative control treatment group and served as the carrier for LUB in the 1.38, 5.5, and 22.0 mg·kg^−1^ treatment groups.

^3^Formulated to contain: 90.60% MgO, 5.05% MnSO_4_, 2.31% CuSO_4_, 1.98% ZnO, 0.03% KI, 0.02% Na_2_SeO_3_, and 0.01% CoSO_4_.

^4^RDP, rumen degradable protein; RUP, rumen undegradable protein. The sum of RDP and RUP is CP. The RDP, RUP, NEm, and NEg were calculated based on the NRC (2000).

Treatments began on day 0 and cattle in each CPE received their respective treatment for 91 d. Emission measurements began at 0800 hours on day 0 and ended at 0500 hours on day 91, immediately preceding cattle removal from the CPE for final BW measurements and transportation to the commercial slaughter facility.

### Cattle pen enclosures

**Figure 1.**
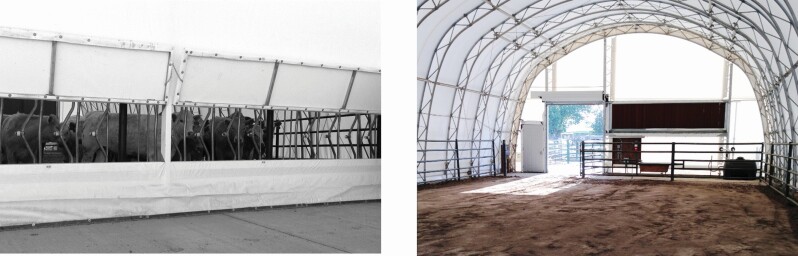
Cattle pen enclosure (CPE).

The CPE were dome shaped, 22.0 × 11.3 m structures oriented east to west, standing 6 m tall at the highest point and constructed with a steel frame, welded truss arches with parallel steel tubes, and continuous structural webbing (11 m Legend Series Cover-All Building, Saskatoon, Saskatchewan, Canada; Figure 1), which was covered with a double stacked Dura-Weave cover (Intertape Polymer Group, Montreal, QC, Canada). Each CPE contained 185 m^2^ of soil surface, 9.1 m linear bunk space on a concrete apron, 3% slope from the bunk to the west of the pen, and a float-activated waterer. Two hinged bunk flaps were used to facilitate feed delivery, and each CPE had two doors. There was one large roll-up door to move cattle in and out of the CPE, and one small door to allow study personnel access to the CPE. Both doors and the bunk flaps remained closed when not in use to prevent disruption of CPE gas equilibrium.

The CPE were thoroughly cleaned before each study cycle began by allowing the existing manure to air dry for 24 to 48 h, and then removing the manure with a skid-loader and power washer. The pen floor was leveled and thoroughly saturated with water to allow volatilization of preexisting NH_3_ from the soil. Fresh soil was applied following a 24-h volatilization period and then compacted with a weighted roller to create a solid pen surface. Accumulation of excreta began on day −7 when cattle were allocated to the CPE and remained uninterrupted for the entirety of the 91-d gas emission measurement period for each cycle.

### CPE airflow and gas measurements

Each CPE was equipped with a 4.9 × 1.2 m cooling pad on the east side for evaporative cooling of incoming ambient air, plus two ventilation fans on the west side to create directional airflow and generate negative pressure inside the CPE. Flow rates were independently determined for all 16 ventilation fans before and after each cycle using a customized purpose-built anemometer and the sum of the two fans within a CPE determined total outflow for each respective CPE. Fan efficiency decay curves were created for the determination of airflow at any given time using the two flow rates obtained at the beginning and end of a cycle. Fan speed was monitored continuously using two sensors (Monarch Instruments, Amherst, NH), and the static differential pressure between internal and external air was monitored to ensure proper ventilation. The temperature (*T*) and relative humidity (**RH**) within CPE were monitored every 15 s during emission sampling periods (Table 2) using RH/T sensors (Dwyer Instruments, Inc., Michigan City, IN), and the same measurements were obtained continuously from ambient air using an on-site weather station (Novalynx, Model 110-WS-16, Auburn, CA).

**Table 2.** Maximum, minimum, and mean daily ambient temperature (TA), relative humidity (RH) and temperature humidity index (THI)^1^

	TA, ºC			RH, %			THI^2^		
Source/cycle	Max.	Min.	Mean	Max.	Min.	Mean	Max.	Min.	Mean
Ambient air									
Cycle 1	42.5	10.1	23.9	70.4	12.9	47.7	86.2	48.6	67.8
Cycle 2	40.0	7.7	21.5	71.3	13.4	52.1	85.9	44.0	65.0
Cycle 3	25.6	0.8	12.2	64.8	18.4	43.2	72.5	31.5	52.7
Cattle pen enclosures									
Cycle 1	40.4	9.0	21.2	94.4	19.5	72.7	85.5	51.9	69.1
Cycle 2	40.0	6.4	19.1	98.7	23.1	79.2	83.3	48.3	66.5
Cycle 3	26.6	-0.8	11.5	99.9	27.5	86.8	70.2	38.7	55.2

^1^The temperature (TA) and relative humidity (RH) within CPE were monitored every 15 s during the 15-min emissions sampling periods using RH/T sensors (Dwyer Instruments, Inc., Michigan City, IN), and the same measurements were obtained for outside ambient air continuously using an on-site weather station (Novalynx, Model 110-WS-16, Auburn, CA).

^2^THI was calculated using the equation of Mader et al. (2006) where THI = (0.8 × TA) + [(RH × 0.01) × (TA – 14.4)] + 46.4; TA = ambient temperature; RH = relative humidity %.

**Figure 2.**
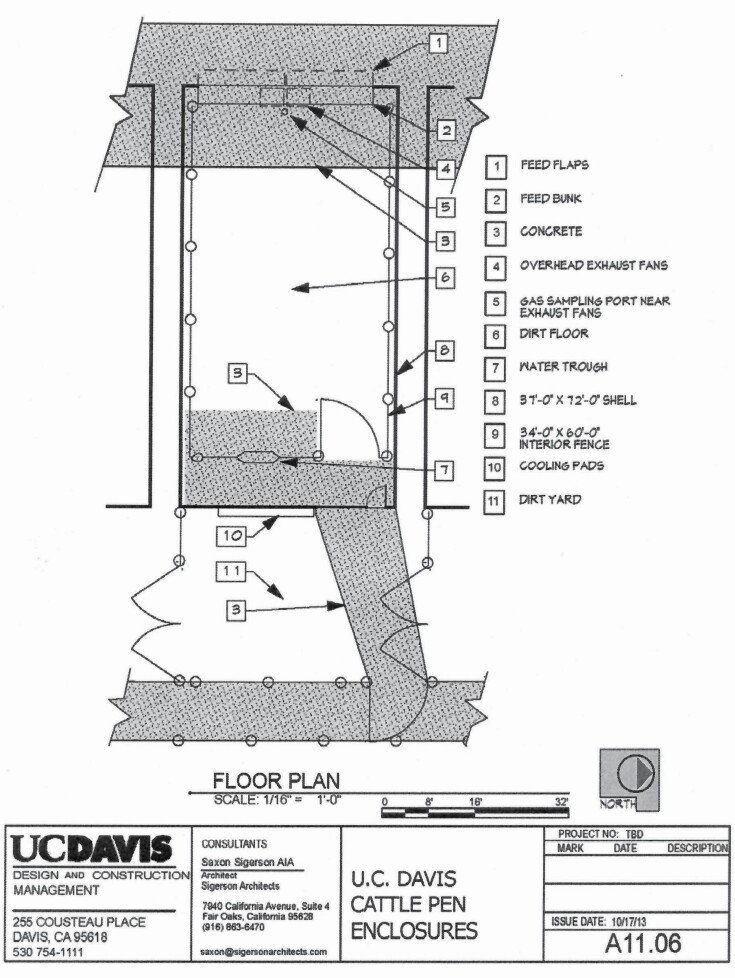
Cattle pen enclosure diagram.

Gas emissions were monitored using calibrated analyzers [Thermo Environmental Instruments (**TEI**), Waltham, MA] for the following five gases: NH_3_ (TEI 17i), CH_4_ (TEI 55c), CO_2_ (TEI 410i), H_2_S (TEI 450i), and N_2_O (TEI 46i). The gas analyzers were located in a temperature-controlled mobile air emissions trailer adjacent to the northernmost CPE, and the inlet to the gas analyzers was independently connected to each of the eight CPE outlets using 103 m of Teflon tubing (9.53 mm OD, 6.35 mm ID) so that gas flowed through the same length of tubing for each CPE being sampled. Gas analyzer outputs were recorded every 15 s using automatic data capture with LabVIEW software (Version 2011, National Instruments, Austin, TX). Gas concentrations were measured from an individual source over 15-min periods in sequential order, starting with ambient air and followed by the eight individual CPE units. The sampling used one stationary sampling port location within each CPE that was adjacent to the outlet fans above the feed bunk (Figure 2), and this procedure was continuous, resulting in a maximum of 11 sampling periods per day for the determination of daily emission rates from a single CPE. Daily emissions were defined as those spanning from 0800 to 0759 hours the next morning, as this time corresponded with disruptions and lag time to gas equilibrium associated with daily feeding and health observations. Sampling was continuous and cycled through the sequence of ambient air followed by the CPE (one to eight) in sequential order. The continuous nature of the emissions monitoring assured that each CPE was sampled around the clock for the duration of the study thereby eliminating potential diurnal bias. In the event a disruption occurred, the sampling sequence began with ambient air followed by the CPE in sequential order.

Before the study, an emissions recovery test was performed for each CPE using purified (99.95%) methane. The average recovery was more than 86% of the expected value. In addition, daily calibration checks were performed to confirm the gas analyzers were functioning properly. A “zero check” was performed to ensure the analyzers read zero when compressed air containing no detectable traces of the gases of interest were introduced. “Span checks” consisted of introducing compressed air with known concentrations of analyte gases and measuring against the analyzer’s calibration specifications. An analyzer was re-calibrated in any instance where either check fell outside specification limits (2% for methane; 10% for all other gases) by introducing a known concentration of analyte gas to the analyzer and adjusting the instrument parameters until the measured value was within the acceptable range.

### Gas data validity

Because gas concentration monitoring was a continuous process, intermittent disruptions to steady-state equilibrium could not be entirely avoided. To account for this, the time and duration of instances where the large door or bunk flaps had to be opened were noted so that the gas measurements during, 5 min before and 15 min after could be identified and removed from the dataset. Entry and exit through the small door did not necessitate the removal of gas data because the airflow disruption during these events was deemed negligible. Gas concentration data compromised because of a gas analyzer failure was handled according to predefined scenarios outlining how replacement data would be substituted. A minimum of 4 min of gas data needed to be available after removal of any required exclusions during a 15-min sampling period to be considered valid. Data representing a minimum of four valid gas sampling periods were required for calculation of daily values. Only one analyzer malfunction occurred throughout the entire study. This malfunction resulted in less than four valid observations for an analyte gas (methane, cycle 1, day 7), for which the mean emissions measured 2 d before and 2 d after (days 5, 6, 8, and 9) the malfunction were substituted for the day 7 data to permit the determination of cumulative emissions.

### Gas emissions calculations

Gas concentrations were measured as the ratio of analyte gas volume to total air volume and were reported in mg/L for CH_4_, CO_2_, and N_2_O, and µg/L for NH_3_ and H_2_S. To calculate gas emission rates for each 15-min sampling period, the concentration of analyte gas in the sample was converted to gram per minute using the molar gas volume in the following equation (equation 1):


$$\begin{array}{ll} & total\;flux\;\left(g/min\right)\;=\\ & \;\left(\frac{\begin{array}{c} (gas\;mg/L\text{-}incoming\;mg/L)\times \\ air\;flow\;(m^{3}/min)\times \\ 1,000\;(L/m^{3}) \end{array}}{\begin{array}{c} (V_{s}\;(L/mol)\times \\ (Temp\;{}^{\circ }C\;+\;273.15)/\\ 273.15\;K) \end{array}}\times MW(g/mol)\right)/1,000,000 \end{array}$$ (1)

where gas mg/L (or µg/L) = gas concentration in the CPE air sample; incoming mg/L (or µg/L) = gas concentration in ambient air; airflow $\left(m^{3}/min\right)$ = airflow rate through the CPE corresponding to the point in time of sampling, calculated as the sum of the individual fan unit airflow rates according to fan efficiency decay curves; $V_{s}\;(L/mol)$ = molar volume of a gas at constant temperature and pressure (both temperature and pressure were held constant in the analyzers, therefore $V_{s}\;(L/mol)$ = 22.4 for all calculations); MW   (g/mol) = molecular weight (MW = 16.04, 44.01, 44.01, 34.08, and 17.03 g for CH_4_, N_2_O, CO_2_, H_2_S, and NH_3_, respectively); and temp = temperature (°C) converted to kelvin (K). To standardize calculated values to g, the denominator was 1,000 times greater for variables measured in microgram per liter than variables measured in milligram per liter (1 million for mg/L, 1 billion for µg/L).

The concentration of analyte gas in ambient air was subtracted from the concentration of analyte gas in samples from each CPE to adjust for baseline values and supply the net amount contributed by the CPE (equation 1). The net concentration was multiplied by the CPE airflow rate and divided by the number of minutes in the sampling period to yield the net emission rate (g/min). The emission rates were then averaged over all sampling periods occurring within defined 24-h periods to produce the daily emission rate (g/min) for individual gases from a CPE. Finally, daily emissions/animal was determined by multiplying the CPE average g/min emission rate by 1,440 min to convert to cumulative daily emissions. Cumulative daily emissions/CPE was then divided by the number of cattle present in the CPE on that day in order to account for removal of cattle during the treatment phase. The resulting daily emission rates (g/animal) were summed over each interim BW measurement period (days 0 to 7, 0 to 14, 0 to 28, and 0 to 56) and over the entire 91-d period to provide cumulative gas emissions, cumulative gas emissions/kg BW, and cumulative gas emissions/kg HCW on a per animal basis.

### Health observations

Cattle were observed daily by trained personnel and abnormal health observations were recorded. Health conditions observed that would deem an animal ineligible or potentially require removal later in the study were documented to prevent affected cattle from being considered for study enrollment. Additional observations were performed by a licensed veterinarian as cattle progressed through the marketing channel, including during loading onto the semi-trailers, during unloading at the abattoir, and finally as ante-mortem observations after a minimum lairage time of 5 h. All cattle euthanized or found dead were necropsied by a veterinarian. During the treatment period, six animals were found dead, and four others were removed from the study due to various conditions.

### Diet formulation and feed assays

Cattle had been fed a concentrate-based diet in a commercial setting before arrival at the study site on day −28, at which point they were provided ad libitum access to water and re-acclimated to a concentrate-based diet using a step-up program involving two intermediate diets based on increasing concentrate levels (~60% and 70%, respectively) and varying proportions of alfalfa and wheat hay. On day −14, cattle were transitioned onto a finishing diet (Table 1) formulated to meet, or exceed, the minimum nutrient requirements for growing beef cattle (NRC, 2000) that was then fed for the remainder of the study. A nonmedicated supplement (i.e., type B feed with a ground corn carrier) was included as 2.5% of the diet DM during acclimation for all cattle from day −14 until the beginning of treatment administration. On day 0, one of four type B supplements were added to the basal diet to provide either 0.0 (CON), 1.38, 5.5, or 22.0 mg·kg^-1^ (DM basis) LUB in a type C (i.e., final feed) medicated feed. All type C feeds containing the appropriate concentrations of LUB or CON were prepared at the study site by adding the same proportions of Type B supplement, water, and basal ration in a rotary mixer wagon (Roto-Mix Forage Express, Dodge City, KS). Mixer procedures (i.e., LUB potency, homogeneity) were validated (i.e., CV ≤ 15%) before study initiation. No concomitant feed additives (ionophores, antibiotics, estrous suppressors, and β-agonists) were used at any point during this study. The mixer wagon was cleaned between each treatment using a flush load comprised of straw and water. Cleaning was also validated to ensure there was no carry-over of LUB between batches. The digital scale on the mixer wagon measured feed deliveries with a 0.45-kg resolution. Prior to use each day, the scale was verified using certified check weights.

Target nutrient densities (% of DM) for CP (13.5%), Ca (0.7%), and P (0.3%) were set based on the recommendations of consulting feedlot nutritionists reported in a survey by Vasconcelos and Galyean (2007). Triplicate samples were collected daily during delivery from the mixer wagon into the bunk for each batch of complete feed and frozen until analysis. Three of the seven composite samples representing a week were randomly selected for each treatment and combined and subsampled for weekly analyses of nutrient content [AOAC methods #985.01 (Ca and P) and 990.03 (CP; Minnesota Valley Testing Laboratories, New Ulm, MN] and LUB concentration (Covance Laboratories, Inc., Greenfield, IN). The minimum acceptable assay value for Ca (0.3%) and P (0.2%) was set to the NRC minimum nutrient requirement for growing beef cattle (NRC, 2000), whereas the minimum acceptable value for CP was set at 12.5%. The threshold for CP was chosen as this was the minimum level recommended by feedlot nutritionists (Vasconcelos and Galyean, 2007). No samples fell below the assay thresholds for CP, Ca, or P.

LUB concentrations were required to be within ± 25% of the target for the 1.38 and 5.5 mg·kg^−1^ samples, and ±20% for the 20 mg·kg^−1^ samples in accordance with FDA guidance (FDA, 2012). The mean LUB potency values for each weekly composite sample over all three study cycles were within the acceptable assay concentration range for each dose level (results not shown). Finally, feed samples from CON were assayed for LUB to confirm the mixer wagon cleaning procedure prevented feeding of LUB, and levels were below the level of quantification (**LOQ** = 0.2 g/ton) in each sample assayed.

### Feeding and growth performance

Individual unshrunk BW measurements were obtained prior to feeding using a certified scale with a 0.45-kg resolution on day −8 (randomization), 0 (initial BW), 7, 14, 28, 56, and 91 d (final BW). Additionally, prior to use the scale was verified with check weights. Feed bunks were assessed daily for each CPE by trained personnel, who estimated orts from the previous day and determined the amount to be provided in a single delivery to ensure ad libitum access to feed. Orts remaining on day 91 were weighed to adjust for refused feed, and DMI (kg∙animal^−1^∙d^−1^) was calculated by dividing the total feed delivery less refused feed by the cumulative number of cattle-days in the CPE to determine as-fed consumption, and then multiplying by diet DM. Thus, cattle were removed from treatment diets a minimum of 24 h before slaughter in order to comply with the Food Use Authorization (FDA, 2016) granted by the FDA. The CPE mean for unshrunk initial and final BW were used to calculate ADG over the 91-d period, and G:F was calculated as a quotient of ADG divided by DMI.

### Slaughter, carcass measurements, and meat quality

On day 91, cattle were loaded onto double-decked aluminum semi-trailers and transported ~1,000 km to a commercial abattoir where they were slaughtered following an ~5 to 9 h lairage. Carcass identification was maintained throughout the slaughter process by recording ear tag sequence at stunning and then cross-matching to sequentially numbered carcass tags. Hot carcass weights and KPH were measured following industry standard processing, and yield grade (**YG**) and quality grade data were collected from left carcass sides by trained university personnel after 22 h in a spray-chill system.

Following chill, striploins (LM) were collected from three randomly selected cattle/CPE and shipped to the University of Illinois Meat Science Laboratory for Warner–Bratzler shear force (**WBSF**) determination. At the laboratory, the anterior end of the striploin was fabricated into 2.54-cm steaks, vacuum packaged, and aged at 4 °C until 14-d postmortem. Steaks were frozen after aging, and then thawed at 4 °C for 24 h before being cooked on a Farberware Open Hearth electric broiler (Farberware, Bronx, NY). Copper-constantan Type-T thermocouples (Omega Engineering, Stamford, CT) connected to a digital scanning thermometer (Barnant Co., Barington, IL) were used to monitor internal temperature, and each steak was flipped a single time when the internal temperature reached 35 °C. The steaks were removed from the grill when a temperature of 70 °C was achieved and cooled to ~25 °C before six cores (1.25 cm diameter) were removed parallel to muscle fiber orientation. Cores were sheared perpendicular to the muscle fibers using a Texture Analyzer TA.HD Plus (Stable Microsystems, Godalming, UK) equipped with a WBSF attachment, and the peak WBSF measurement was averaged over all 6 cores to obtain a single shear force measurement (kg of force) for each steak.

### Statistical analysis

Data were analyzed using version 9.2 of SAS (SAS Institute, Cary, NC), and the individual CPE were considered the experimental unit. Continuous variables were analyzed using PROC MIXED, with treatment (LUB dose), sex, and the dose × sex interaction as fixed effects, and cycle as the random effect included in the model. If the dose × sex interaction was not significant (*P* > 0.05), the main effect of dose pooled across sexes was evaluated. When the main effect of dose was significant (*P* ≤ 0.05) or tended to be significant (0.05 ≤ *P* ≤ 0.10), planned contrasts comparing each LUB dose to CON were performed in a pairwise fashion. If the dose × sex was significant (*P* ≤ 0.05) planned contrasts comparing each LUB dose to CON within sex were performed in a pairwise fashion. Treatment means were estimated using the LSMEANS statement.

Prior to study conduct, the CVM concurred that for the claim variables (gas emissions per unit of BW or HCW) the dose range for each variable would include those dosages significantly different from CON. Additionally, methodology to determine the minimum effective dosage and maximum effective dosage was agreed upon.

The minimum effective dose for the claim variables was determined to be the smallest dose used in the study that differed from CON based on the planned contrasts performed following a significant *F*-test (*P* < 0.05). To determine the lowest maximum effective dose, a dose–response curve fit to the least squares means of the doses was performed. If the dose–response curve was determined to be a linear plateau model (Anderson and Nelson, 1975) and the slope or slopes were different (*P* ≤ 0.05) from zero, then the maximum effective dosage was the “join point” where the plateau began. The join point was identified by assessing five specific, competing linear and linear plateau models based on the smallest *P*-value indicating best fit: (i) Linear = linear from 0 to 22.0 mg·kg^−1^ DM; (ii) Quadratic1 = linear from 0 to 1.38 mg·kg^−1^ DM, plateau from 1.38 to 22.0 mg·kg^−1^ DM; (iii) Quadratic2 = linear from 0 to 5.5 mg·kg^−1^ DM, plateau from 5.5 to 22.0 mg·kg^−1^ DM; (iv) Quadratic3 = no response from 0 to 1.38 mg·kg^−1^ DM, but linear from 1.38 to 5.5 mg·kg^−1^, plateau from 5.5 to 22.0 mg·kg^−1^ DM; and (v) Quadratic4 = no response from 0 to 1.38 mg·kg^−1^, and linear from 1.38 to 22.0 mg·kg^−1^ DM.

Discrete variables were analyzed with a generalized linear mixed model using a binomial distribution and logit link function in PROC GLIMMIX. The classification of fixed and random effects and the handling of interactions and pairwise comparisons were performed in a similar manner as the continuous variables. Statistical analyses for YG were performed on both continuous and discrete (YG 1 = 1.00 to 1.99, YG 2 = 2.00 to 2.99, and so on) forms of data, and quality grades were further sorted into the five categories routinely used for determining premium or discount adjustments when cattle are marketed on a grid-based system (U.S. Prime, upper 2/3 Choice, low Choice, Select, and Standard). Because the model did not converge due to sparseness of data for mortality, YG 4, and Select and Prime quality grades, Fisher’s exact test was performed using PROC FREQ to evaluate the frequency distribution of the CON cattle compared with LUB. Statistical significance for the main effects of dose was determined by *P* ≤ 0.05 and tendencies were declared when 0.05 ≤ *P* ≤ 0.10.

## Results

There were no interactions observed between dose and sex for any variable measured in the study (*P* ≥ 0.063). Therefore, the main effects of dose (Tables 3, 4, and 6) and sex are presented (Tables 7 and 8).

**Table 3.** Least squares means for the effect of lubabegron (LUB) dose on cumulative gas emissions and cumulative gas emissions standardized by final BW and HCW for five gases measured from cattle pen enclosures (CPE) over 91 d^1^

	LUB (mg·kg^−1^ DM)						Dose	Significance of contrast		
Variable	0.0	1.38	5.5	22.0	SEM	Dose × sex *P*-value^2^	*P*-value^2^	Control vs. 1.38 mg·kg^−1^	Control vs. 5.5 mg·kg^−1^	Control vs. 22.0 mg·kg^−1^
Unshrunk final BW, kg^3^	567	583	582	582	19.4	0.916	0.257			
HCW, kg	349	364	365	365	11.7	0.865	0.035	0.019	0.014	0.014
NH_3_										
Total emissions, g/animal	7,783	7,093	6,860	6,751	855	0.281	0.052	0.076	0.023	0.013
Standardized by BW, g/kg	13.6	12.1	11.7	11.6	1.19	0.161	0.004	0.009	0.002	<0.001
Standardized by HCW, g/kg	22.3	19.5	18.7	18.5	1.97	0.147	0.001	0.004	<0.001	<0.001
CH_4_										
Total emissions, g/animal	10,466	10,692	10,763	10,476	638	0.712	0.895	—	—	—
Standardized by BW, g/kg	18.4	18.3	18.5	18.0	1.06	0.439	0.858	—	—	—
Standardized by HCW, g/kg	30.0	29.3	29.5	28.7	1.81	0.376	0.601	—	—	—
CO_2_										
Total emissions, kg/animal	720	758	734	755	47.3	0.616	0.302	—	—	—
Standardized by BW, g/kg	1,268	1,299	1,261	1,299	61.3	0.322	0.268	—	—	—
Standardized by HCW, g/kg	2,061	2,081	2,013	2,070	107.8	0.269	0.331	—	—	—
H_2_S										
Total emissions, g/animal	20.6	19.9	20.3	20.0	6.04	0.581	0.975	—	—	—
Standardized by BW, g/kg	0.035	0.033	0.035	0.033	0.0110	0.417	0.905	—	—	—
Standardized by HCW, g/kg	0.057	0.055	0.055	0.055	0.0176	0.379	0.776	—	—	—
N_2_O^4^										
Total emissions, g/animal	−27.4	−36.8	−36.4	−34.7	10.2	0.279	0.627	—	—	—
Standardized by BW, g/kg	−0.046	−0.062	−0.060	−0.060	0.0154	0.306	0.693	—	—	—
Standardized by HCW, g/kg	−0.075	−0.097	−0.095	−0.095	0.0264	0.311	0.742	—	—	—

^1^Emissions were measured from an individual CPE (*n* = 8) over 15-min sampling periods using calibrated, gas-specific analyzers (Thermo Environmental Instruments (TEI), Waltham, MA). This procedure was continuous so that a maximum of 11 sampling periods were available for determination of daily emission rates/animal for each CPE. Emissions were measured from 0800 hours on day 1 until 0500 hours on day 91.

^2^If the dose × sex interaction was not significant (*P* > 0.05), contrasts were constructed to evaluate the main effect of dose for the pooled sexes. Statistical significance for dose was declared when *P* ≤ 0.05 and tendencies were declared when 0.05 < *P* ≤ 0.10.

^3^Unshrunk; initial BW did not differ by dose (*P* = 0.937; Table 6).

^4^Measured values from CPE were less than the values reported from ambient air, resulting in negative values.

**Table 4.** Least squares means for the effect of lubabegron (LUB) on cumulative NH_3_ gas emissions and standardized cumulative NH_3_ gas emissions corresponding to each BW measurement^1^

	LUB (mg·kg^−1^ DM)						Dose	Significance of contrast		
Variable	0.0	1.38	5.5	22.0	SEM	Dose × sex *P*-value^2^	*P*-value^2^	Control vs. 1.38 mg·kg^−1^	Control vs. 5.5 mg·kg^−1^	Control vs. 22.0 mg·kg^−1^
Unshrunk BW, kg										
Initial	451	454	455	452	10.0	0.996	0.937			
Day 7	464	469	470	469	10.4	0.968	0.935			
Day 14	474	484	484	478	12.3	0.962	0.655			
Day 28	496	508	506	501	12.2	0.990	0.627			
Day 56	529	545	542	539	15.2	0.974	0.411			
Final	567	583	582	582	19.4	0.916	0.257			
NH_3_, g										
Days 0 to 7	415	394	387	326	49	0.746	0.022	0.439	0.300	0.004
Days 0 to 14	953	835	801	699	106	0.802	0.006	0.062	0.020	< 0.001
Days 0 to 28	2,097	1,783	1,686	1,563	265	0.573	0.006	0.027	0.006	< 0.001
Days 0 to 56	4,619	4,089	3,888	3,763	540	0.420	0.019	0.050	0.011	0.004
Days 0 to 91	7,783	7,093	6,860	6,751	855	0.281	0.052	0.076	0.023	0.013
NH_3_, g/kg BW										
Days 0 to 7	0.89	0.84	0.82	0.70	0.106	0.619	0.003	0.278	0.139	<0.001
Days 0 to 14	2.01	1.73	1.66	1.47	0.223	0.698	<0.001	0.010	0.002	<0.001
Days 0 to 28	4.21	3.53	3.33	3.13	0.509	0.463	<0.001	0.004	<0.001	<0.001
Days 0 to 56	8.66	7.50	7.14	6.99	0.871	0.258	<0.001	0.004	<0.001	<0.001
Days 0 to 91	13.6	12.2	11.7	11.6	1.19	0.161	0.004	0.009	0.002	<0.001

^1^Emissions were measured from an individual CPE (*n* = 8) over 15-min sampling periods using calibrated, gas-specific analyzers (Thermo Environmental Instruments (TEI), Waltham, MA). This procedure was continuous so that a maximum of 11 sampling periods were available for determination of daily emission rates/animal for each CPE. Daily gas emissions were defined as those within the span beginning at 0800 hours and ending at 0759 hours the next morning. Cumulative emissions were standardized by the BW measured on the day corresponding to the emissions time-period for reporting.

^2^If the dose × sex interaction was not significant (*P* > 0.05), contrasts were constructed to evaluate the main effect of dose for the pooled sexes. Statistical significance for dose was declared when *P* ≤ 0.05 and tendencies were declared when 0.05 < *P* ≤ 0.10.

**Table 5.** Statistical significance of five linear and linear plateau models used to determine the minimum effective and lowest maximum effective lubabegron (LUB) doses for reducing ammonia (NH_3_) gas emissions per kilogram of body weight and hot carcass weight over the entire 91-d period^1^

	Model^2^				
Variable	1	2	3	4	5
NH_3_/kg BW, *P*-values	0.0107	0.0005	0.0004	0.0033	0.0052
NH_3_/kg HCW, *P*-values	0.0056	0.0002	0.0001	0.0015	0.0025

^1^The minimum effective dose for NH_3_ gas emissions per kg BW and HCW was determined to be the smallest dose used in the study that differed from the control based on planned contrasts performed following a significant *F*-test (*P* < 0.05). To determine the lowest maximum effective dose, a dose–response curve fit to the least squares means of the doses was performed. If the dose–response curve was determined to be a linear plateau model (Anderson and Nelson, 1975) and the slope or slopes were different (*P* ≤ 0.05) from 0, then the maximum effective dosage was the “join point” where the plateau began.

^2^Five competing linear and linear plateau models were evaluated based on the smallest *P*-value indicating best fit: (i) Linear = linear from 0 to 22.0 mg·kg^−1^ DM; (ii) Quadratic1 = linear from 0 to 1.38 mg·kg^−1^ DM, plateau from 1.38 to 22.0 mg·kg^−1^ DM; (iii) Quadratic2 = linear from 0 to 5.5 mg·kg^−1^ DM, plateau from 5.5 to 22.0 mg·kg^−1^ DM; (iv) Quadratic3 = no response from 0 to 1.38 mg·kg^−1^ DM, linear from 1.38 mg·kg^−1^ DM to 5.5 mg·kg^−1^ DM, plateau from 5.5 to 22.0 mg·kg^−1^ DM; and (v) Quadratic4 = no response from 0 to 1.38 mg·kg^−1^ DM, linear from 1.38 to 22.0 mg·kg^−1^ DM.

**Table 6.** Least squares means for the effect of lubabegron (LUB) on growth performance traits and carcass characteristics of beef cattle over a 91-d treatment period

	LUB (mg·kg^−1^ DM)					Dose × sex	Dose	Significance of contrast		
Variable	0.0	1.38	5.5	22.0	SEM	*P*-value^1^	*P*-value^1^	Control vs. 1.38 mg·kg^−1^	Control vs. 5.5 mg·kg^−1^	Control vs. 22.0 mg·kg^−1^
Growth performance^2^										
Unshrunk initial BW, kg	451	454	455	452	10.0	0.996	0.937			
Unshrunk final BW, kg	567	583	582	582	19.4	0.916	0.257			
DMI, kg	8.8	9.2	8.8	8.8	0.56	0.980	0.585			
ADG, kg	1.27	1.42	1.39	1.43	0.126	0.724	0.075	0.027	0.067	0.023
G:F, kg:kg	0.144	0.156	0.158	0.163	0.0071	0.856	0.031	0.065	0.033	0.005
Carcass characteristics										
HCW, kg	349	364	365	365	11.7	0.865	0.035	0.019	0.014	0.014
Dressing percentage^2^	61.5	62.4	62.7	62.8	0.34	0.738	0.002	0.006	<0.001	<0.001
Adjusted fat thickness, cm	1.28	1.15	1.24	1.19	0.119	0.994	0.579			
LM area, cm^2^	88.4	94.8	96.1	96.8	1.78	0.063	<0.001	<0.001	<0.001	<0.001
Marbling score^3^	623	573	560	562	16.7	0.979	0.058	0.051	0.018	0.022
Calculated yield grade^4^	2.68	2.37	2.41	2.25	0.164	0.816	0.155			
KPH, %	1.96	1.99	1.96	1.61	0.066	0.802	0.002	0.742	0.981	0.001
Lean maturity^5^	162	163	167	163	3.5	0.731	0.262			
Skeletal maturity^5^	172	170	170	172	1.9	0.585	0.592			
Overall maturity^5^	169	168	169	168	2.5	0.639	0.805			
14-d WBSF^6^, kg	2.48	2.79	2.92	2.75	0.117	0.620	0.017	0.022	0.003	0.039

^1^If the dose × sex interaction was not significant (*P* > 0.05), contrasts were constructed to evaluate the main effect of dose for the pooled sexes. Statistical significance for dose was declared when *P* ≤ 0.05 and tendencies were declared when 0.05 < *P* ≤ 0.10.

^2^Growth performance and dressing percentage were based on unshrunk initial and final BW.

^3^500, Small^00^; 600, Modest^00^.

^4^Yield Grade = 2.50 + (0.98 × adj. fat thickness, cm) + (0.2 × KPH, %) + (0.0084 × HCW, kg) – (0.05 × LM, cm^2^) (USDA, 1997).

^5^100, A^0^ maturity; 200, B^0^ maturity.

^6^WBSF, Warner–Bratzler shear force, measured after a 14-d aging period.

**Table 7.** Least squares means for the effect of sex on growth performance, carcass characteristics, and NH_3_ gas emissions in beef cattle over a 91-d period

	Sex			Dose × sex	Sex
Variable	Steers	Heifers	SEM	*P*-value^1^	*P*-value^1^
Growth performance^2^					
Unshrunk initial BW, kg	475	432	9.2	0.996	<0.001
Unshrunk final BW, kg	601	556	18.9	0.916	<0.001
DMI, kg	9.1	8.6	0.53	0.980	0.038
ADG, kg	1.38	1.38	0.12	0.724	0.875
G:F, kg:kg	0.151	0.159	0.0065	0.856	0.064
Carcass characteristics					
HCW, kg	376	346	11.3	0.865	<0.001
Dressing percentage^2^	62.5	62.2	0.30	0.738	0.078
Adjusted fat thickness, cm	1.13	1.30	0.110	0.994	0.024
LM area, cm^2^	14.7	14.5	0.26	0.063	0.216
Marbling score^3^	567	592	11.8	0.979	0.156
Calculated yield grade^4^	2.39	2.46	0.137	0.816	0.582
KPH, %	1.65	2.10	0.049	0.802	<0.001
Lean maturity^5^	164	164	3.3	0.731	0.854
Skeletal maturity^5^	167	175	1.7	0.585	<0.001
Overall maturity^5^	166	171	2.4	0.639	<0.001
14-d WBSF^6^, kg	2.80	2.67	0.100	0.620	0.142

^1^If the dose × sex interaction was not significant (*P* > 0.05), the main effect of sex was evaluated for the pooled doses. Statistical significance for sex was declared when *P* ≤ 0.05 and tendencies were declared when 0.05 ≤ *P* ≤ 0.10.

^2^Growth performance and dressing percentage were based on unshrunk initial and final BW.

^3^500, Small^00^; 600, Modest^00^.

^4^Yield Grade = 2.50 + (0.98 × adj. fat thickness, cm) + (0.2 × KPH, %) + (0.0084 × HCW, kg) – (0.05 × LM, cm^2^) (USDA, 1997).

^5^100, A^0^ maturity; 200, B^0^ maturity.

^6^WBSF, Warner–Bratzler shear force, measured after a 14-d aging period.

**Table 8.** Least squares means for the effect of sex on cumulative gas emissions and cumulative gas emissions standardized by final BW and HCW for five gases from cattle measured over 91 d^1^

Variable	Sex		SEM	Dose × sex	Sex
	Steers	Heifers		*P*-value^1^	*P*-value^1^
Unshrunk final BW, kg	601	556	18.9	0.916	<0.001
HCW, kg	376	346	11.3	0.865	<0.001
NH_3_					
Total emissions, g/animal	7,264	6,979	835.3	0.281	0.283
Standardized by BW, g/kg	12.0	12.5	1.16	0.161	0.198
Standardized by HCW, g/kg	19.3	20.1	1.75	0.147	0.153
CH_4_					
Total emissions, g/animal	11,169	10,029	591.3	0.712	0.005
Standardized by BW, g/kg	18.6	18.1	1.02	0.439	0.200
Standardized by HCW, g/kg	29.7	29.1	1.75	0.376	0.298
CO_2_					
Total emissions, g/animal	764,608	719,176	46,084.4	0.616	0.010
Standardized by BW, g/kg	1,271	1,293	60.2	0.322	0.216
Standardized by HCW, g/kg	2,033	2,080	106.0	0.269	0.109
H_2_S					
Total emissions, g/animal	21.5	18.9	5.96	0.581	0.066
Standardized by BW, g/kg	0.035	0.034	0.0101	0.417	0.385
Standardized by HCW, g/kg	0.057	0.054	0.0164	0.379	0.430
N_2_O					
Total emissions, g/animal	−42.5	−25.1	9.35	0.279	0.008
Standardized by BW, g/kg	−0.070	−0.044	0.0146	0.306	0.017
Standardized by HCW, g/kg	−0.112	−0.070	0.0235	0.311	0.018

^1^Emissions were measured from an individual CPE (*n* = 8) over 15-min sampling periods using calibrated, gas-specific analyzers (Thermo Environmental Instruments (TEI), Waltham, MA). This procedure was continuous so that a maximum of 11 sampling periods were available for determination of daily emission rates/animal for each CPE. Emissions were measured from 0800 hours on day 1 until 0500 hours on day 91.

^2^If the dose × sex interaction was not significant (*P* > 0.05), the main effect of sex was evaluated for the pooled doses. Statistical significance for sex was declared when *P* ≤ 0.05 and tendencies were declared when 0.05 < *P* ≤ 0.10.

### Mortality

The number of mortalities during the treatment phase was 3, 1, 0, and 2 for CON, 1.38, 5.5, and 22.0 mg·kg^−1^ treated cattle, respectively, and was similar (*P* ≥ 0.246) across treatments (results not shown). Necropsy findings suggested ruminal acidosis was the likely cause of death in four of the six mortalities, and these cases were spread across treatments (1, 1, 0, and 2 cattle for CON, 1.38, 5.5, and 22.0 mg·kg^−1^, respectively). The remaining two mortalities were both CON cattle, with the etiologies being unknown for one animal and interstitial pneumonia for the other. An additional four cattle (two CON cattle and two from the 22.0 mg·kg^−1^ group) were removed from the study during the treatment phase, with three removed due to various degrees of musculoskeletal injury and one due to bloat complications. No cattle were withdrawn at any point during shipment for slaughter, and all cattle passed USDA ante-mortem inspection following lairage at the abattoir.

### Dose effects

#### Gas emissions

Dose had no effect (*P* ≥ 0.268) on cumulative emissions or cumulative emissions standardized by BW or HCW for CH_4_, CO_2_, N_2_O, or H_2_S during any interim time period (results not shown) or for the entire 91-d treatment period (Table 3). However, there was an effect of LUB on NH_3_ gas whereby reductions in cumulative NH_3_ gas emissions tended (*P* = 0.052) to be affected by LUB, with gas emissions from cattle fed 22.0 mg·kg^−1^ being 13.3% less (*P* = 0.013) than those of the CON cattle compared to a reduction (*P* ≤ 0.076) of only 8.9 and 11.9% for the 1.38 and 5.5 mg·kg^−1^ LUB groups, respectively. Correspondingly, LUB reduced (*P* ≤ 0.009) NH_3_ gas emissions/kg BW and HCW vs. CON. The magnitude of the reduction in NH_3_ gas emissions/kg BW achieved by feeding 1.38, 5.5 and 22.0 mg·kg^−1^ LUB over the 91-d period was 11.0%, 14.0%, and 14.7%, respectively. For NH_3_ gas emissions/kg HCW, the magnitude of reductions in response to feeding 1.38, 5.5, and 22.0 mg·kg^−1^ LUB over the 91-d period were 12.6%, 16.1%, and 17.0%, respectively.

LUB affected cumulative NH_3_ gas emissions (*P* ≤ 0.022) and NH_3_ gas emissions/kg BW (*P* ≤ 0.003) in each of the four time periods corresponding to interim BW measurements (Table 4). From days 0 to 7, cumulative NH_3_ gas emissions and NH_3_ gas emissions/kg BW were 21.4% and 21.7% lower (*P* ≤ 0.004) for cattle fed 22.0 mg·kg^−1^ LUB compared with CON, respectively, but neither the cumulative NH_3_ gas emissions nor standardized gas emissions for cattle fed 1.38 or 5.5 mg·kg^−1^ LUB differed from CON (*P* ≥ 0.139). However, NH_3_ gas emissions/kg BW were lower (*P* ≤ 0.010) for cattle fed LUB vs. the CON cattle during days 0 to 14, 0 to 28, and 0 to 56, regardless of dose. Cumulative NH_3_ gas emissions from days 0 to 14 were 15.9% and 26.7% lower for the cattle fed 5.5 and 22.0 mg·kg^−1^ LUB vs. CON (*P* ≤ 0.020), respectively, whereas the emissions from cattle fed 1.38 mg·kg^−1^ only tended (*P* = 0.062) to be reduced vs. CON during this period. From days 0 to 28 and 0 to 56, cattle fed LUB had ≥11.5% reductions (*P* ≤ 0.050) in NH_3_ gas emissions compared with CON, regardless of dose.

The minimum effective dose for NH_3_ gas emissions per kilogram BW and HCW was determined to be the smallest dose used in the study that differed from CON based on planned contrasts performed following a significant *F*-test (*P* < 0.05). The maximum effective dose was determined to be 5.5 mg/kg because the model assuming a linear response from 0 to 5.5 mg·kg^−1^ DM and then a plateau from 5.5 to 22.0 mg·kg^−1^ DM (Quadratic2, Table 5) had the best fit of the five models evaluated for both NH_3_ gas emissions/kg BW and NH_3_ gas emissions/kg HCW. Therefore, the minimum effective dose and lowest maximum effective dosage were determined to be 1.38 and 5.5 mg·kg^−1^ DM, respectively.

#### Growth performance and carcass characteristics

Initial BW did not differ (*P* = 0.937) among LUB treatments, and there was no effect (*P* = 0.585) of LUB on DMI (Table 6). Compared with cattle receiving CON, G:F was increased (*P* ≤ 0.065) by 8.3%, 9.7%, and 13.2% for cattle fed 1.38, 5.5, and 22.0 mg·kg^−1^ LUB, respectively. The effect of LUB treatment on G:F was reflected by a tendency to improve (*P* = 0.075) ADG, with ADG increased (*P* ≤ 0.067) vs. CON by 11.8%, 9.4%, and 12.6% in cattle fed 1.38, 5.5 and 22.0 mg·kg^−1^ of LUB, respectively; yet final BW was not altered by LUB treatment (*P* = 0.257).

Compared with CON cattle, cattle fed LUB had carcass weights ~15 kg heavier (*P* ≤ 0.035), dressing percentages 0.9 to 1.3 units greater (*P* ≤ 0.006), and LM areas 6.4 to 8.4 cm^2^ larger (*P* ≤ 0.001; Table 6). Adjusted fat thickness was not affected (*P* = 0.579) by LUB treatment. Compared with carcasses from CON cattle, KPH was only reduced (*P* = 0.001) when cattle were fed 22.0 mg·kg^−1^ LUB (1.96% vs. 1.61%; Table 6). LUB treatment had no effect (*P* = 0.155) on calculated YG. When YG was analyzed as a discrete variable, the probability of cattle producing a YG3 carcass was greater (*P* < 0.019) for CON- than LUB-fed cattle (Figure 2); the probability of cattle producing YG1, YG2, or YG4 carcasses was similar (*P* ≥ 0.233) across treatments.

**Figure 3.**
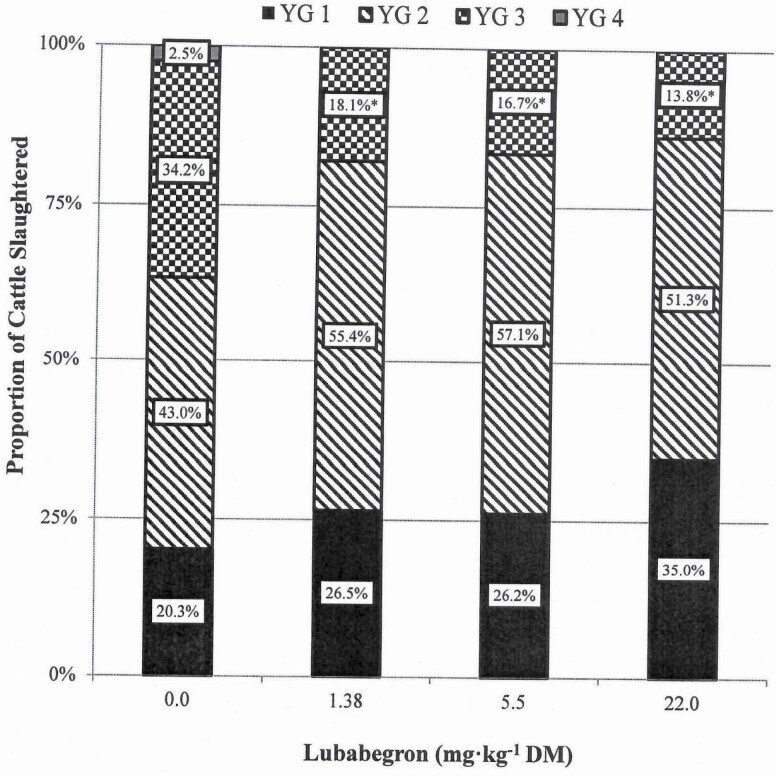
Discrete yield grade (YG) expressed as a proportion of the cattle slaughtered within each treatment. Within a YG category, means for the non-zero LUB treatment groups marked with an “*” differ from the control (*P* ≤ 0.05). Values represented in this figure are arithmetic means, whereas the denoted differences are between the least squares means calculated using PROC GLIMMIX and represent the probability of cattle in a pen displaying a given response.

LUB treatment had no effect (*P* ≥ 0.262) on skeletal, lean, or overall maturity. Marbling scores tended (*P* = 0.058) to be influenced by LUB treatment with carcasses of LUB-fed cattle having marbling scores 50 to 63 points less (*P* ≤ 0.051) than CON carcasses (Table 6). Feeding LUB shifted the quality grade distribution lower, whereby cattle fed LUB had a lower (*P* = 0.004) probability of grading high Choice and a greater (*P* = 0.021) probability of grading low Choice than CON (Figure 3). Dark cutter incidence was not influenced by treatment, as only a single carcass fell into this category over the entire study. WBSF was 0.27 to 0.44 kg greater (*P* ≤ 0.039) for striploins from LUB-treated cattle than CON cattle (Table 6).

### Sex effects

**Figure 4.**
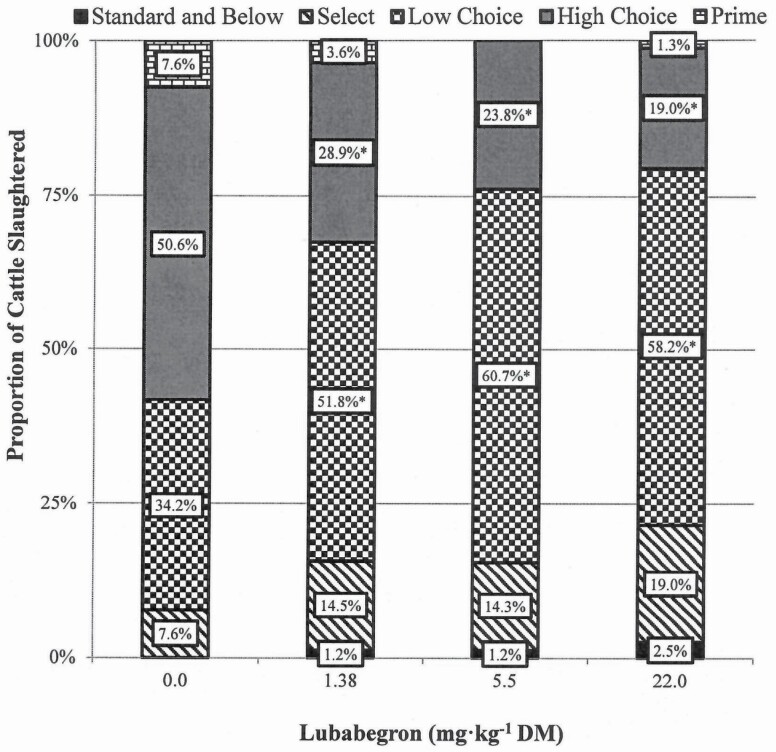
Quality grade expressed as a proportion of the cattle slaughtered within each treatment. Within a quality grade, means for the non-zero LUB treatment groups marked with an “*” differ from the control (*P* ≤ 0.05). Values represented in this figure are arithmetic means, whereas the denoted differences are between the least squares means calculated using PROC GLIMMIX and represent the probability of cattle in a pen displaying a given response.

Steers started the treatment period 43 kg heavier (*P* < 0.001) than heifers. During the 91-d period, steers consumed 0.5 kg/d more DM (*P* = 0.038) than heifers. Both sexes gained weight similarly (*P* = 0.875), resulting in heifers tending to have 5% greater G:F (*P* = 0.064) than steers (Table 7). Heifers produced carcasses that were 30 kg lighter (*P* < 0.001) than steers with 1.17 cm greater (*P* = 0.024) adjusted fat thickness and 0.45 % units greater (*P* < 0.001) KPH; LM area and calculated YG did not differ (*P* > 0.216) between steers and heifers. Lean maturity was not affected (*P* > 0.854) by sex, but heifers had marginally higher (*P* < 0.001) skeletal maturity (A^75^ vs. A^67^) than steers. Marbling score and WBSF did not differ (*P* > 0.142) between steers and heifers (Figure 4).

No measures of NH_3_ emissions (cumulative or weight standardized) differed (*P* > 0.153) between steers and heifers (Table 8). Total emissions of CH_4_ and CO_2_ were ~6% to 11% greater (*P* < 0.10) for steers compared to heifers. However, when standardized by BW and HCW, no differences (*P* > 0.109) were noted for CH_4_ and CO_2_ between the sexes. There was a tendency for steers to emit more (*P* < 0.066) H_2_S than heifers, but this sex effect was also mitigated (*P* > 0.385) when standardized by weight.

## Discussion

The NRC (2003) report on air emissions from animal feeding operations identified NH_3_ as having the greatest relative importance of pollutants on a global, national, and regional scale. The National Oceanic and Atmospheric Administration (NOAA, 2014) and the EPA (EPA, 2004) also recognize NH_3_ as an important pollutant involved with the deterioration of ecosystems, reduced visibility, and reductions in air quality due to formation of fine particulate matter created by reactions with nitric and sulfuric acid. Fine particulate matter, which refers to particles with a mean aerodynamic diameter <2.5 µm (**PM**_**2.5**_), affects more humans than any other pollutant monitored, and can be hazardous to health due to the ability of the particles to infiltrate pulmonary bronchioles and impair alveolar gas exchange (Gauderman et al., 2004).

A 2008 ruling by the EPA exempted Concentrated Animal Feeding Operations (**CAFO**) from having to report NH_3_ gas emissions under the Comprehensive Environmental Response, Compensation, and Liability Act (**CERCLA**), but ruled feedlots with permitted capacities greater than 1,000 animals and daily NH_3_ gas emissions surpassing 45.5 kg were required to report under the Emergency Planning and Community Right-To-Know Act (**EPCRA**; Waldrip et al., 2015). However, a ruling in 2013 by the U.S. Court of Appeals for the District of Columbia Circuit deemed NH_3_ could be presumptively regulated under the Clean Air Act due to the precursory role of NH_3_ to the formation of PM_2.5_ (EPA, 2016). As a result, a subsequent ruling in 2017 vacated the 2008 CAFO exemptions, potentially making livestock operations of any size subject to the reporting requirements outlined in CERCLA and EPCRA (*Waterkeeper Alliance et al. v. U.S. EPA, 2017*; U.S. Court of Appeals, 2017). In 2019, however, the EPA amended the release notification regulations under the EPCRA to add the reporting exemption for air emissions from animal waste at farms (Federal Register, 2019).

### Previous research investigating strategies to mitigate NH_3_ gas emissions from feedlots

Ammonia emissions from CAFOs are primarily the result of microbial hydrolysis of urinary urea nitrogen (**UUN**) by fecal bacteria containing a urease enzyme that produces carbon dioxide and ammonium, which can then volatize to NH_3_ gas as excreta pH alkalizes (Cole et al., 2005; Archibeque et al., 2007; Vasconcelos et al., 2007). Previous research suggests feedlot cattle only retain a small portion (10% to 20%) of the nitrogen (**N**) consumed, whereas the majority is excreted as UUN (Cole et al., 2006; Koenig and Beauchemin, 2013; Waldrip et al., 2015), and the quantity of UUN excreted depends on factors such as protein degradability of the diet consumed and the nutrient requirements of the animal (Cole et al., 2005; Vasconcelos et al., 2007; Archibeque et al., 2007). Furthermore, the relative importance of NH_3_ gas emissions from feedlots continues to be of significant interest due to the expansion of the ethanol industry and availability of high-protein byproducts for use as a low-cost feedstuff. Feeding greater proportions of these feedstuffs increases the quantity of N excreted, which subsequently becomes susceptible to volatilization as NH_3_ gas (Cole et al., 2005; Hales et al., 2012; Hünerberg et al., 2013).

Although CP concentrations of the finishing diet fed in the current study were based on the recommendations reported in a 2007 survey of feedlot nutritionists, it should be noted these recommendations remain relatively unchanged according to the respondents of a more recent version of the same survey (Samuelson et al., 2016). It is generally understood that the metabolizable protein requirements of feedlot cattle are not static throughout a feeding period; but rather, these requirements generally decrease as cattle mature and the composition of gain shifts from predominately protein deposition early in the feeding period to primarily fat closer to harvest (NRC, 2000). When a static CP concentration is fed, the efficiency of N utilization, as a function of intake, is inherently reduced and more N is excreted late in the feeding period, thereby increasing potential NH_3_ losses (Cole et al., 2005; Vasconcelos et al., 2007, 2009). Vasconcelos et al. (2009) reported that the proportion of dietary N that was retained in the body decreased as CP concentration and length of the feeding period increased for crossbred steers, and that there was a linear increase in fecal and urinary N excretion as dietary CP concentration and length of the feeding period increased. As such, it is rational that the exploration of methods aimed to improve N use efficiency and to mitigate N excretion as cattle mature has served as the cornerstone for research designed to mitigate NH_3_ gas emissions from feedlots.

As potential strategies to reduce NH_3_ gas emissions, precision and phase-feeding programs geared toward feeding lowered CP concentrations that satisfy the requirements needed for optimal performance during different phases of the growth curve have been evaluated. Vasconcelos et al. (2009) reported that excretion of UUN was increased when greater percentages (14.5% vs. 13.0% vs. 11.5%) of dietary CP were fed to crossbred steers. Using data from the same study, Cole et al. (2005) noted in vitro NH_3_ gas emissions were increased 60% to 200% after 30, 75, and 120 d from cattle fed diets targeted to contain 13.0% vs. 11.5% CP. As a second treatment level, Cole et al. (2005) also evaluated three different urea inclusion rates to determine the effect of N degradability on in vitro NH_3_ gas emissions. Although N degradability did not interact with dietary CP, greater urea concentrations did increase NH_3_ gas emitted over a 7-d span. More recently, Koenig et al. (2013) used a passive horizontal flux sampling technique to measure NH_3_ gas emissions from pens of crossbred steers fed barley diets containing 12.6% or 14.0% CP, and observed a numeric reduction of nearly 50% in NH_3_ gas in each of five different periods when emissions were collected over 4 d. When expressed as a fraction of N intake, NH_3_ gas emissions were 40% less for cattle fed the lower level of CP, although the fraction emitted from either treatment (7.8% vs. 12.7%) was considerably lower than what has been reported previously in the literature (Hristov et al., 2011; Waldrip et al., 2015). The wide range of NH_3_ gas emission rates found in the literature may be partially explained by the different approaches used to quantify NH_3_ gas emissions in open feedlots vs. laboratory or closed-chamber settings, in addition to the spatial variation of NH_3_ gas concentrations (Hristov et al., 2011).

While an opportunity exists to reduce NH_3_ gas emissions from feedlot cattle through the use of phase and precision-feeding programs, the adoption of these practices remains minimal because of logistical challenges and availability of high-protein ethanol byproducts. In practical terms, reducing NH_3_ gas by lowering dietary CP is difficult because most of the supplemental CP fed is urea, and urea is needed as a source of degradable intake N to optimize organic matter fermentation by bacteria in the rumen (NASEM, 2016). Milton et al. (1997) and Shain et al. (1998) reported a 4% to 8% increase in ADG of crossbred steers when urea was added to a diet predominately comprised of dry-rolled corn. Likewise, Cole et al. (2006) evaluated phase-feeding programs with steam-flaked corn diets and reported that removing urea during the last 56 d on feed reduced ADG by almost 7% compared with cattle fed 13.0% CP for the entire feeding period. When looking solely at the final 56 d of the same study, which corresponded to the timing of changes in CP inclusion for the phase-feeding treatment, the ADG of the cattle that continued to receive 13.0% CP was 8.5% and 16.3% greater compared with cattle whose urea concentrations were reduced so that their total dietary CP was 11.5% and 10.0% CP, respectively. However, cattle switched to the lower levels of CP also had lower DMI over that same time period compared with those fed a static level of CP, which is noteworthy as this may also have contributed to differences in BW gain and underlines the need to consider unintended consequences when altering diet composition in feedlots. Albeit a different source of supplemental N, Archibeque et al. (2007) described a roughly 25% reduction in G:F and 20-kg lighter beef carcasses when cattle were fed diets formulated to contain 9.1% CP compared with diets containing soy bean meal formulated to 11.8% or 13.9% CP. Collectively, inadvertent shortcomings in these findings are especially relevant if trying to reduce NH_3_ gas emissions, as meeting protein requirements or hindering the extent of ruminal organic matter fermentation would have a detrimental effect because reductions in growth performance and longer feeding periods required to achieve a desired endpoint would inevitably increase NH_3_ gas emissions.

### An alternative approach

To date, life cycle assessments have been the primary means for evaluating the effect of growth-promoting technologies on the environmental impact of livestock production systems, and typically focus on reducing resources (e.g., feedstuffs, water, and land) or maximizing productivity (e.g., growth rate and slaughter weight) while holding the other constant (Pelletier et al., 2010; Capper, 2011; Capper and Hayes, 2012). A recent meta-analysis of life cycle assessments encompassing 742 food production systems by Clark and Tilman (2017) suggested increasing resource efficiency through use of conventional systems would be more advantageous for the environment than switching to nonconventional systems such as organic agriculture or grass-fed beef. A few studies have employed an approach similar to the current study and outlined the environmental benefit of various technologies by collecting an array of emissions measurements (Cooprider et al., 2011; Stackhouse-Lawson et al., 2013). However, LUB (Experior; Elanco, Greenfield, IN) is the first technology with a clinical effectiveness program designed to support an indication for a reduction in gas emissions.

LUB reduced NH_3_ gas emissions/kg of unshrunk BW and HCW when fed at a dose as low as 1.38 mg·kg^−1^ of DM over the 91-d feeding period in the current study. No additional reduction in NH_3_ gas emissions was observed in cattle fed 22.0 mg·kg^−1^ compared with cattle fed the 5.5 mg·kg^−1^ dosage [the inclusion rate range approved by the FDA is 1.4 to 5.5 mg·kg^−1^ DM; full information regarding the label can be referenced in the Freedom of Information summary (FDA, 2018)]. There are three avenues which can result in reductions in NH_3_ gas/kg BW and HCW: (i) reduced NH_3_ gas emissions, (ii) increased BW and/or HCW, or (iii) a combination of both. The reductions in NH_3_ gas emissions/kg BW or HCW for cattle fed LUB were driven by both a decrease in NH_3_ gas emissions and increased BW and HCW. The quantitative reduction of 8.9% to 13.3% in NH_3_ gas emissions of LUB relative to CON cattle observed in concert with a 4.6% increase in HCW provides evidence that LUB works on both sides of the ratio. From this observation, some general modes of action of LUB can be hypothesized. First, it is unlikely that the reduction in NH_3_ gas by cattle fed LUB was a function of reduced N intake. Dietary CP was constant across treatments due to the common diet being fed, and feed intake was equivalent for LUB- and CON-fed cattle. The most likely explanation to describe how LUB reduces NH_3_ gas emissions while concurrently improving growth performance is to consider a greater retention of nutrients within the body. Since protein comprises an average of 16.5% of retail beef cuts (Benedict and Ellis, 1987; Heinz and Hautzinger, 2007) and protein is 16% N, it is reasonable to hypothesize that a greater magnitude of protein accretion in the carcass would result in more N being captured and, thus, not available to be excreted as UUN, with subsequent volatilization as NH_3_ gas. A major source of the additional N deposited into beef carcass can be derived from reduced NH_3_ gas emissions since ammonia is 82.2% N. For example, the amount of N conserved from every 1,000 g of reduced ammonia gas emission is equivalent to the amount of N in 5,138 g of protein or 31.1 kg of beef carcass. For the present study, cumulative ammonia gas reduction was 690, 923, and 1,032 g·animal^−1^ for 1.4, 5.5, and 22 mg·kg^−1^ DM LUB, respectively. Increased HCW for each of the respective LUB treatments was 15, 16, and 16 kg·animal^−1^. The theoretical efficiency of N conservation was 69.9%, 55.7%, and 49.9% for 1.4, 5.5, and 22 mg·kg^−1^ DM LUB, respectively.

In addition to the increase in carcass weight discussed above, evaluation of other carcass characteristics may provide further information on the physiological effects of LUB. Routine measurements of carcass fat showed no change to adjusted fat thickness, a decrease in KPH at only the highest LUB dose, and a reduction in marbling score for all doses of LUB. Minimal data from LUB-treated cattle are available for comparison, but Hwang et al. (2021) characterized β-adrenergic receptors in bovine intramuscular and subcutaneous adipose tissue, and those results provide relevant information regarding the mechanism by which adipose tissue may be affected by the β _3_ agonist/β _1,2_ antagonist, LUB. The relative mRNA expression reported for β _1_, β _2_, and β _3_ adrenergic receptors was greater in subcutaneous than intramuscular adipose tissue, and the β _2_ adrenergic receptor subtype was the most abundant mRNA in both tissues. There was minimal mRNA expression for the β _1_ and β _3_ receptors in intramuscular fat, and the authors concluded this could likely limit the response to agonists of these receptor subtypes. Results from Hwang et al. (2021) suggest the decrease in marbling score observed in the current study was likely not due to lipolysis. To fully elucidate how marbling score was reduced by LUB, evaluation of chemical composition and adipose cellularity would be needed, but the dilution of intramuscular fat by LM hypertrophy has been offered as a mechanism by which marbling score is reduced by other biotechnologies that increase LM area (Duckett et al., 1999; Gonzales et al., 2020). In other words, increases to LM area without a corresponding increase to intramuscular fat would result in a decrease in marbling score without any changes to lipogenesis or lipolysis. In the current study, cattle that received LUB had a 7% to 10% increase in LM area and a corresponding 8% to 10% decrease in marbling score. Future evaluations of adipocytes and additional observations comparing changes in LM area and marbling score would further the understanding of LUB effects on intramuscular fat. This clinical registration study was designed to supplement LUB for the maximum labeled duration of 91 d; the impact of shorter durations on marbling score is currently unknown. Exploring the impact of duration on marbling score could determine if this is a means to mitigate depression of marbling score and corresponding shifts to quality grade.

Cattle that received LUB for 91 d produced LM steaks with mean 14-d WBSF values that were 0.27 to 0.44 kg greater than that of CON. Little comparative data are available for shear force of steaks from LUB-fed cattle, however, increases in shear force are not unexpected as similar effects have been reported for other biotechnologies that alter muscle development (Garmen and Miller, 2014). Despite an increase in WBSF, mean shear force values of steaks from LUB-treated cattle in the current study (2.75 to 2.92 kg) were within the range of recent North American surveys that report WBSF for top loin steaks (~2.0 to 3.4 kg: Guelker et al., 2013; Howard et al., 2013; Igo et al., 2015; Martinez et al., 2017). The influence of changes in shear force on consumer acceptability have been reported to vary depending on where within the range of WBSF observations occur (Platter et al., 2003), thus additional evaluations of shear force for LUB-fed cattle would further the understanding of this effect. The present study measured shear force at a single postmortem aging period of 14 d, however, the most recent National Beef Tenderness Survey (Martinez et al., 2017) reported post-fabrication aging time for boneless striploins or toploins was 27.2 d for retail stores and 34.6 d for foodservice operations (Martinez et al., 2017). Future studies investigating the interactive effects of postmortem aging and LUB supplementation would be valuable.

## Conclusion

The findings of this study indicate that LUB effectively reduces NH_3_ gas emissions/kg of unshrunk final BW and HCW from feedlot cattle when fed at a dose as low as 1.38 mg·kg^−1^ DM over a 91-d period. Feeding LUB at a dose >5.5 mg·kg^−1^ of DM did not result in additional reduction of NH_3_ gas/kg BW or HCW. LUB had no effect on cumulative emissions or cumulative emissions standardized by BW or HCW for CH_4_, CO_2_, N_2_O, or H_2_S.

## Abbreviations

CP: crude protein
CPE: cattle pen enclosure(s)
KPH: kidney, pelvic, and heart (percentage of fat)
LM: longissimus dorsi muscle
LUB: lubabegron
PM_2.5_: particulate matter <2.5 µm
RH: relative humidity
SβM: selective beta modulator
UUN: urinary urea nitrogen
WBSF: Warner–Bratzler shear force
YG: yield grade
